# Exercise, brain plasticity, and depression

**DOI:** 10.1111/cns.13385

**Published:** 2020-06-03

**Authors:** Jin‐Lei Zhao, Wan‐Ting Jiang, Xing Wang, Zhi‐Dong Cai, Zu‐Hong Liu, Guo‐Rong Liu

**Affiliations:** ^1^ School of Physical Education and Health Shanghai Lixin University of Accounting and Finance Shanghai China; ^2^ Department of Physical Education and Sport Training Shanghai University of Sport Shanghai China

**Keywords:** brain function, brain plasticity, brain structure, depression, exercise

## Abstract

Depression is a common mental disorder characterized by high incidence, high disability, and high fatality, causing great burden to the society, families, and individuals. The changes in brain plasticity may be a main reason for depression. Recent studies have shown that exercise plays a positive role in depression, but systematic and comprehensive studies are lacking on brain plasticity changes in depression. To further understand the antidepressive effect of exercise and the changes in brain plasticity, we retrieved related literatures using key words “depression,” “depressive disorder,” “exercise,” “brain plasticity,” “brain structure,” and “brain function” from the database of Web of Science, PubMed, EBSCO host, and CNKI, hoping to provide evidence for exercise in preventing and treating depression. Increase in exercise has been found negatively correlated with the risk of depression. Randomized controlled experiments have shown that aerobic exercise, resistance exercise, and mind‐body exercise can improve depressive symptoms and levels. The intensity and long‐term effect of exercise are now topical research issues. Exercise has been proven to reshape the brain structure of depression patients, activate the function of related brain areas, promote behavioral adaptation changes, and maintain the integrity of hippocampal and white matter volume, thus improving the brain neuroprocessing and delaying cognitive degradation in depression patients. Future studies are urgently needed to establish accurate exercise prescriptions for improving depressive symptoms, and studies on different depressive populations and studies using multimodal brain imaging combined with multiple analytical methods are also needed.

## 1 INTRODUCTION

Depression is a common mental disorder with high morbidity, disability, and mortality. According to the World Health Organization (WHO), there are about 350 million people suffering from depression worldwide. By 2020, depression will be the second largest disease after heart disease, causing a great burden to society, families, and individuals.^1^ At present, drug therapy is the main treatment of depression, but it is hindered by side effects, addiction, high price, and poor patient compliance. Drug treatment is overall not satisfactory, with the quality of life of patients being seriously affected.

Studies have confirmed that, as a nondrug way, exercise can help alleviate depressive symptoms, with comparable efficiency to drug therapy and other psychological interventions.^2^, ^3^ Exercise can also prevent other diseases, such as diabetes, osteoporosis, and obesity.^4^, ^5^ However, there have been different conclusions on the effect of different exercises on depression. Recently, more studies have shown structural and functional changes in certain parts of the central nervous system, which is the anatomical basis of depression.^6^, ^7^, ^8^ Brain plasticity refers to the ability of brain to change its structure and function, which is also the physiological basis for psychological and behavioral adaptation.^9^ With the advances in medical imaging, the changes in brain plasticity of depression patients have attracted wide attention. Researchers have done more studies at the molecular, cellular, behavioral, and structural network levels. However, there have been no systematic and comprehensive conclusions on brain plasticity change in exercise‐induced antidepression.

Here, we summarized the effects of exercise on depression, analyzed different exercise modes in depressive people, and expounded the changes in brain plasticity in exercise‐induced antidepression, hoping to provide more information on exercise‐induced antidepression and brain plasticity theory, finally contributing to the design of precise exercise prescriptions for depression.

## 2 EVIDENCE OF EXERCISE EFFECT ON DEPRESSION

### 2.1 Observational study

Exercise can not only enhance physical health, reduce diseases, but also promote psychological development. Salgureo et al^10^ found that physical activity was significantly correlated with depression in 436 elderly Spanish people (60‐98 years) (the Geriatric Depression Scale, GDS score), and more active physical activity was associated with lower depression level. Another study with 622 elderly people showed that low‐intensity physical activity (<150 min/wk) increased the risk of depression (OR = 4.23) and led to lower cognitive function.^11^ In a meta‐analysis, Schuch et al^12^ found that people with high levels of physical activity were less likely to suffer from depression (OR = 0.83), which was more prominent in the elderly. In addition, lower limb muscle strength, balance, and walking speed were found negatively correlated with depression level in the elderly, indicating that the motor function of the elderly was closely related to depression.^13^

Long‐term follow‐up studies further revealed the association between exercise and depression. A recent 11‐year follow‐up study of 33 908 adults found that regular exercise has helped to reduce depression, with 1‐hour exercise a week reducing the risk of depression by 12%.^2^ After 2 years of follow‐up, Li et al^14^ found that every 10 MET‐min/d decrease in physical activity level increased the risk of depression by 1.1% in boys and 2.1% in girls among college students, and every 10‐s prolongation in running test increased the risk of depression by 1.5% in boys and 6.3% in girls; thus, the author believed that cardiopulmonary endurance played a mediating role in physical activity and depression risk. Improving physical activity and cardiopulmonary endurance can effectively reduce the risk of depression. Physical activity can also reduce the disability rate of depressed elderly. Lee and Park's study followed 645 depressed elderly people over 65 years old for 1 year, and found that physical activity reduced the functional disability and alleviated the symptoms of depression. Therefore, proper physical activity is necessary for those elderly people.^15^

Different exercises have different effects on depression. The intensity and duration of exercise have independent correlation with depression. Epidemiological studies have confirmed that aerobic or resistance exercises such as ball games, jogging, cycling, dancing, swimming, and Taijiquan have antidepressant effects. Chen et al^16^ investigated the frequency, duration, and intensity of exercise in 2724 elderly people with depression in Taiwan, China. Only exercise intensity had independent correlation with depression level. Exercise energy consumption of about 2000 kcal per week can effectively reduce the risk of depression in the elderly. While another survey of 2006 elderly people in Korea showed that exercise intensity, duration, and frequency were significantly correlated with the depressive symptoms.^17^ Hamer and Stamatakis^18^ followed up 6359 elderly people for 2 years and found that moderate‐intensity exercise at least once a week slowed down depression and improved speech fluency and memory.

### 2.2 The research on experimental intervention

Cumulative randomized controlled trials and a number of systematic reviews have explored the impact of exercise on depression. Aerobic exercise, resistance exercise, and mind‐body exercise were the most common exercise interventions. As early as 1984, McCann and his colleagues investigated the effect of aerobic exercise on depression. Forty‐three depression patients were randomly assigned to an aerobic exercise treatment group, a relaxation therapy group, and a no‐treatment group, and the results provided the first controlled evidence concerning the effects of aerobic exercise on depression.^19^ Later, the interventions of aerobic exercise, such as walking and jogging, were found to improve the symptoms of depression.^20^ Researchers also found that resistance exercise can be used alone or as an adjuvant treatment for depression. In 1997, Singh and his colleagues randomized 32 older adults with depression to either a resistance exercise group or a control group, and they found depression was significantly improved after resistance exercise.^21^ In recent years, Taijiquan and Yoga, which can be regarded as mind‐body exercise, have been accepted and loved by many people to relieve depression symptoms.^22^, ^23^ Here, we reviewed the antidepression effects of these three different types of exercise: aerobic exercise, resistance exercise, and mind‐body exercise (Table 1).

**Table 1:** Effect of different exercise modes on depression

Reference	Participant	Age (y)	Gender	Diagnosis	Exercise prescription	Main outcome
*Aerobic exercise*						
Knubben et al^29^	20	49 ± 13	Male/female	DSM‐IV BRMS	Walk for 10 d, 5 times a day, 80% THR, 15 min	BRMS↓, CES‐D↓
Blumenthal et al^30^	51	≥40	Male/female	DSM‐IV BDI‐II	Jogging, 16 wk, 3 times a week, 70%‐85% HRR intensity, 45 min	HAMD↓
Helgadóttir et al^31^	620	18 ~ 67	Male/female	PHQ‐9	Fitness exercises, stretching and balance exercises, 12 wk, 3 times a week, intensity 60%‐80% MHR, 55 min	MADRS↓
Hanssen et al^32^	34	37.8	Male/female	ICD‐10 BDI‐II	Cycling, 4 wk, 3 times a week, 60%‐80% VO_2max_ intensity, 35 min, 30 s interval	BDI‐II ↓
Trivedi et al^33^	122	18 ~ 70	Male/female	DSM‐IV HDRS	Running + cycling, 12 wk, 2‐3 times a week, 4‐16 KKW intensity	HDRS↓
*Resistance exercise*						
Lecheminant et al^38^	30	26.9 ± 5.1	Female	/	Resistance movement of instruments, 18 wk, twice per week, 8‐12 twice per group, 3 groups, 90‐s interval	CES‐D↓
Chin et al^39^	41	81.0 ± 5.8	Male/female	/	Instrument resistance movement, 24 wk, twice a week, 8‐12 twice a group, 2 groups, 45‐60 min	GDS (constant)
Chen et al^40^	65	≥65	Male/female	/	Elastic exercise with resistance, 64 wk, 3 times a week, 40 min	CSDD↓ ··
Aidar et al^41^	11	51.7 ± 8.0	Male/female	/	Antiresistance movement of instruments, 12 wk, 3 times a week, 8‐10 times a group, 3 groups, 50%1RM intensity, 60‐min practice, 2‐min interval	BDI↓·
Singh et al^42^	60	≥60	Male/female	DSM‐IV GDS	Antiresistance movement of instruments, 8 wk, 3 times a week, 8 times a group, 3 groups, intensity 20%‐80%1RM, exercise for 60 min	GDS↓ HDRS↓
Khorvash et al^43^	60	25.1 ± 3.2	Male/female	Beck and Kettle questionnaire	Antiresistance movement of the instrument, 10 wk, twice a week, a total of 20 times, 90 min	Beck and Kettle questionnaire↓
*Mind‐body exercise*						
Kinser et al^49^	15	40.93 ± 15.84	Female	MINI	Hatha Yoga, 8 wk, once a week, intensity based on the difficulty of posture, practice 75 min	PHQ‐9↓
Kinser et al^50^	15	40.93 ± 15.84	Female	MINI	Hatha Yoga, 8 wk, 1 wk, intensity based on the difficulty of posture, 75 min; 1‐year follow‐up	PHQ‐9↓
Uebelacker et al^51^	63	46.78 ± 12.27	Male/female	DSM‐IV QIDS	Hatha Yoga, 10 weeks, twice a week, 80‐min	QIDS↓ PHQ‐9↓
Prathikanti et al^52^	20	22 ~ 72	Male/female	MINI MMSE BDI‐II	Hatha Yoga, 8 wk, twice a week, 90 min	BDI‐II ↓
Yeung et al^53^	23	18 ~ 70	Male/female	DSM‐IV HDRS	Yang Taijiquan, 12 wk, twice a week, 60 min	BDI‐II↓, HDRS↓
Li et al^54^	30	38 ~ 76	Male/female	HAMD	Sitting Taijiquan, 5 wk, twice a week, 30 min	HAMD↓
Lavretskyet al.^56^	33	69.1 ± 7.0	Male/female	MMSE HDRS	Taijiquan, 10 wk, once a week, 120 min	HDRS↓

Abbreviations: ↑, going up; ↓, going down; BDI‐II, Beck Depression Inventory‐II; BRMS, Bech‐Rafaelsen Melancholia Scale; CES‐D, Center for Epidemiologic Studies‐Depression; CSDD, Cornell Scale for Depression; DSM‐IV, Diagnostic and Statistical Manual of Mental Disorders; GDS, the Geriatric Depression Scale; HAMD, HDRS, Hamilton Depression Scale; HRR, heart rate reserve; ICD‐10, International Classification of diseases; KKW, kcal/kg/week; MADRS, Montgomery and Asberg Depression Rating Scale; MHR, maximum heart rate; MINI, Mini‐International Neuropsychiatric Interview; MMSE, Mini‐Mental State Examination; PHQ‐9, Patient Health Questionnaire‐9; QIDS, Quick Inventory of Depressive Symptomatology—Self‐Report; THR, target heart rate.

#### 2.2.1 Aerobic exercise

Aerobic exercise is easy to engage and has great health benefits, and it is based on aerobic metabolism, big muscle group, long‐lasting time, and regular rhythm.^24^ Many researches have revealed good antidepressant effect of aerobic exercise. Aerobic exercise can change monoamine neurotransmitters, increasing the levels of 5‐HT and norepinephrine and reducing the cortisol level, leading to alleviation of depressive symptoms.^25^ In addition, aerobic exercise was also associated with neuroactive substance concentration in the central nervous system of depressive rats and the activation of brain BDNF.^26^ Active aerobic exercises also increase beta‐endorphin.^27^

Although many studies have shown that aerobic exercise has a better antidepressant effect than traditional medicine, the dose‐response to aerobic exercise in people with depression remains equivocal. Systematic reviews have shown that moderate‐intensity aerobic exercise for at least 9 weeks, 3‐4 days a week, can effectively reduce the risk of depression.^28^ Previous studies have shown that long‐term exercise appears to be more effective as compared to short‐term one. Nevertheless, one study showed that aerobic interval training intensity of 80% of maximum heart rate (MHR) in a short period of training (10 days) could substantially improve symptoms of depression.^29^ Blumenthal and colleagues found that aerobic exercise with intensity of 70%‐85% MHR, 30‐minute sessions, three times a week for four months, had a similar effect with antidepressant.^30^ Helgadttir et al^31^ found that the Montgomery and Asberg Depression Rating Scale (MADRS) scores of depressive patients (18‐67 years) were significantly lower after low‐intensity aerobic exercise compared with moderate‐ or high‐intensity aerobic exercises (three times a week for 12 weeks); therefore, the author believed that moderate‐ and high‐intensity aerobic exercises are more effective than the low‐intensity one. Another study reported on short‐term (4 weeks, three times a week), high‐intensity (80% VO_2max_), and low‐intensity (60% VO_2max_) bicycling exercises in patients with unipolar depression, and found that the Beck Depression Inventory‐II (BDI‐II) score was decreased by 85% after high‐intensity exercise.^32^ Trivedi et al^33^ explored the effects of 12‐week high‐intensity walking (16 kcal/kg/wk) and low‐intensity walking (4 kcal/kg/wk) on depression, and the Hamilton Depression Scale (HDRS) showed that both of them achieved significant improvement in depression (*P* < .001), and intensive exercise was more conducive to reducing depression levels.

Current evidence indicates that high‐intensity aerobic exercise is superior to the low‐intensity one for depression treatment. Due to different forms of aerobic exercise and individual differences, the methods to evaluate exercise intensity are different between studies. There are absolute index (METs) and relative index (%HRR, %HRmax,%VO2max), and the ranges of low‐, moderate‐, and high‐intensity exercise are also different, which makes it hard to provide significant insight into the effective exercise intensity for depression treatment.^34^ So more accurate and rigorous limits are needed for aerobic exercise intensity in depression treatment, and systematic studies should be conducted to explore the dose‐response effect of aerobic exercise intensity in depressive people.

#### 2.2.2 Resistance exercise

Resistance exercise is characterized by muscle against resistance, and it is an effective way to increase muscle strength, volume, and endurance. Resistance exercise can not only delay muscular degeneration, promote metabolism, and effectively reduce age‐related falls and fractures, but also alleviate anxiety, inferiority, and other bad moods.^35^, ^36^ Compared with aerobic exercise, resistance exercise has been less studied for its role in depression treatment, but evidence was found that resistance exercise can be used separately or jointly for depression treatment.^37^

Lecheminant et al^38^ gave progressive resistance exercises (18 weeks and twice a week) to 30 postpartum depressive women. The subjects chose nine major muscle groups to exercise according to their own exercise intensity (1‐3 groups, 8‐1 twice a group, 90 seconds between groups). The results showed that the CES‐D was decreased significantly in the resistance exercise group, with increased self‐efficacy of the participants (*P* = .016). However, some other researches made inconsistent conclusions. Chin et al^39^ randomly divided 173 elderly people into the resistance exercise group, functional exercise group, combined exercise group, and control group, and resistance exercise group received 45‐ to 60‐minutes moderate‐intensity training twice a week—the GDS showed that neither resistance exercise nor functional training could significantly improve the depression (*P* > .05).

Progressive exercise is widely used for studying resistance exercise intervention in depression. Chen et al^40^ used elastic band resistance exercise to improve depressive symptoms in patients with Alzheimer's disease. Sixty‐five elderly people (65 years) were trained for 15 months (three times a week, 40 minutes), and the Cornell Scale for Depression (CSDD) score was decreased after exercise, with significant improvement found in lower limb dysfunction and sleep disorders (*P* < .05). Aidar et al^41^ reported that 11 patients with ischemic stroke were given strength exercises of the upper and lower limbs (12 weeks, three times a week, 50%1RM); they found that the BDI score was decreased after exercise (*P* = .021) and the exercise intensity was negatively correlated with depression risk. Singh et al^42^ compared the effects of 8‐week high‐intensity (80%1RM) and low‐intensity (20%1RM) resistance exercise (three times per week, 60 minutes) on mild depression in the elderly; they found that the GDS and HDRS scores were reduced by 61% and 29% in the two groups (*P* < .14). Participants receiving high‐intensity exercise had higher compliance, indicating a better antidepressant effect compared with low‐intensity exercise.

We noticed that the antidepressant effect of resistance exercise may be through regulating monoamine transmitters and neuroimmunological indicators. Khorvash et al^43^ reported that resistive exercise for 10 weeks (twice a week for 90 minutes) effectively improved the depressive symptoms (*P* < .001) and decreased C‐reactive protein in 60 depressive college students. Other studies found that after 8 weeks of strength training (three times per week, 50%‐70%1RM), the CES‐D scores of depressive patients were decreased, the plasma levels of 5‐HT and the NE were increased, and the cortisol levels were decreased. Therefore, the change in monoamine transmitter is associated with exercise‐induced improvement in depression.

Although resistance exercise has been proven to have antidepression effect, it is more difficult to implement resistance exercise in actual exercise plan than aerobic exercise. Resistance exercise requires high skill guidance and almost perfect equipment, which is a potential obstacle, and long‐term follow‐up studies and detailed descriptions of intensity and type of exercise are still needed for resistance exercise.

#### 2.2.3 Mind‐body exercise

Yoga and Taijiquan are well accepted and accessible, and they emphasize the integration of body, spirit, and external environment, improving the overall health through slow body movement, deep breathing, and meditation. They are also known as mind‐body exercise. Mind‐body exercise can help to reduce negative emotion, relieve fatigue, improve sleep quality, and prevent cardiovascular and cerebrovascular diseases.^44^, ^45^, ^46^ Growing studies have shown that mind‐body exercise can alleviate the depression symptoms.

A meta‐analysis of 12 randomized controlled trials found that yoga had significantly better antidepressant effects than routine care, relaxation, and aerobic exercise.^47^ Another systematic review indicated similar short‐term effects between yoga and antidepressants for depressive symptoms.^48^ Recently, the long‐term effect of yoga against depression has attracted wide attention. Kinser et al^49^ recruited 15 female patients with depression for 8‐week Hatha Yoga training (once a week, 75 minutes). The control group was given health education, and they found that the scores of Patient Health Questionnaire‐9 (PHQ‐9) were decreased in both groups. One‐year follow‐up found significantly improved depression in patients involved in long‐term regular yoga (*P* < .05).^50^ Another latest research found no significant difference in moderately depressed women between 10‐week yoga group and health education group (*P* = .36), but after 6 months, the PHQ‐9 score was decreased by more than 50% in 51% of the women in the yoga group.^51^ Notably, the subjects of the above studies all had moderate or severe depression; Prathikanti et al^52^ found that BDI‐II scale scores were decreased more significantly in moderate and mild depression patients receiving 8‐week yoga than those receiving attention exercises (*P* = .034).

Yeung et al^53^ observed the effect of Yang's Taijiquan in patients with depression, and found that the HAMD score of patients was reduced by 50% (*P* < .05) after 12 weeks (twice per week, 60 minutes). They believed that Taijiquan could improve the symptoms of patients with depression. Taijiquan is often used in patients with poststroke depression. Through rhythmic movements, Taijiquan can help patients improve their lost neuromuscular function and treat their depression from psychological and physiological aspects. Li et al^54^ gave sitting Tai Chi (wheelchair Taijiquan) to patients with poststroke depression for 5 weeks, and found greater improvement in the experimental group than in the conventional treatment group. Another study also showed that traditional 6‐style Taijiquan exercise (8 weeks, 30 minutes each) could improve depression and the score of HAMD in patients with poststroke depression. The author recommended Taijiquan throughout the rehabilitation of poststroke depression.^55^ In addition, the antidepressant effect of Taijiquan combined with drugs is better than that of drug therapy alone. Lavretsky et al^56^ gave escitalopram to 112 sixty‐year‐old patients with depression and divided them into Taijiquan group (20 standard movements, 10 times, once a week) and health education group. The results showed that the escitalopram combined with Taijiquan decreased HDRS score and significantly improved the cognitive function of depression patients, suggesting a synergistic effect between Taijiquan and antidepressants in improving depressive symptoms.

## 3 EFFECTS OF EXERCISE ON BRAIN PLASTICITY OF DEPRESSION PATIENTS

### 3.1 Changes in brain plasticity of depression patients

Increasingly more evidence revealed that depression is closely related to brain structure and functional changes. Functional magnetic resonance imaging (fMRI), event‐related potentials (ERP), and spontaneous electroencephalograms (EEG) have identified structural and functional abnormalities in key brain regions of depression patients, including volume changes and functional damage.^57^ Techniques such as voxel‐based morphometry (VBM) and near‐infrared spectroscopy (NIRS) have led to the availability of many useful methods for further identifying brain plasticity changes in depression patients.

#### 3.1.1 Changes in brain structure

In patients with depression, brain structure changes are closely associated with certain parts of the nervous system, including the frontal lobe, cingulate gyrus, hippocampus, striatum, and white matter.^58^ Reductions in brain volume (including structural brain changes such as neuronal loss and decreased neurotrophic factor) are related to depressive episodes. The hippocampus plays an important role in cognitive activity, as well as stress and mood regulation in patients with depression. Some studies have shown visibly reduced hippocampal volumes and abnormal emotional regulation in patients with depression_._ ^59^, ^60^ Further, a recent meta‐analysis of 15 fMRI‐based studies found that patients with depression had decreased hippocampal volumes, with particularly significant decreases in patients with early‐onset depression (<21 years), which may possibly prolong the depression course and increase the frequencies of relapse. Indeed, the authors believed that chronic stress caused an increase in glucocorticoid levels, accompanied by hypothalamic‐pituitary‐adrenal axis dysfunction, hippocampal structure atrophy, and decreased neurogenesis, ultimately leading to depression.^57^ Depression in adult patients may be related to destruction of synaptic connections between hippocampal neurons. An autopsy study found that patients with depression showed impaired plasticity of hippocampal neurons, manifested as a decrease in hippocampal gray matter density, and reduction in nerve fiber network and hippocampal neurogenesis. Reduction in hippocampal volume also occurs in elderly patients with depression.^61^

Examination of abnormal structure and function of the prefrontal lobe is important to study brain plasticity in patients with depression. Studies have shown that prefrontal injury is accompanied by a marked abnormality of emotional regulation and control. An autopsy study showed decreased nerve cell volume and reduced glial cell density in the prefrontal cortex of depression patients.^62^ A meta‐analysis by Bora et al^63^ including 23 VBM‐based studies showed reduced volume of the prefrontal cortex region (the anterior cingulate, orbital frontal cortex, and dorsolateral frontal cortex) in depression patients. In addition, patients with depression also have extensive microstructural abnormalities, characterized by damage to the white matter fiber tracts such as the frontal lobe, parietal lobe, and temporal lobe.^64^ Peng and Qiu et al^65^, ^66^ observed the frontal cortex in first‐episode treatment‐naïve patients with depression, and they found that the thicknesses of the temporal pole, right orbital frontal gyrus, and paracentral region were increased. Besides, there were also changes in the surface area of the frontal cortex. Zhao et al^67^ found that cortical thickness change was mainly found in the prefrontal cortex‐limbic system of first‐episode treatment‐naïve patients. These new variables can help explain the neuropathological process of depression at an early stage.

#### 3.1.2 Changes in brain function

There are a wide range of brain dysfunction and asymmetry in the patients with depression during the resting state. Resting‐state functional magnetic resonance imaging（RS‐fMRI）data of depressed patients showed increased ReHo values of the left hippocampus, bilateral parahippocampal gyrus, left middle temporal gyrus, and caudate nucleus, and decreased ReHo values of the right middle temporal gyrus, right inferior temporal gyrus, and right cerebellum. Abnormal spontaneous neuronal activity was observed in multiple brain regions at resting state in depression patients.^68^ Kenny et al^69^ found that in elderly depressed people, the regions with greater connectivity (*P* ≤ .05) included the anterior central gyrus, middle frontal gyrus, paracentral lobule, thalamus, and lingulate, and other areas, which were related to thought and attention. Studies also showed a close correlation between asymmetry of resting frontal EEG and depressive symptoms. Evidence showed that EEG data of depression patients had lower activation of the left prefrontal cortex than that of healthy people, indicating that the functional activity of dorsolateral prefrontal cortex, especially the left dorsolateral prefrontal cortex, might play a major role in depression progression.^70^, ^71^ The experimental design of resting brain function changes is simple and easy to control, and only few factors can affect the analysis results, which is of great significance for diagnosis and efficacy evaluation of depression.

The brain dysfunction of depression patients is also reflected by the changes in neurological activity when performing cognitive tasks. Lv et al^72^ assigned the Oddball task to patients with first‐episode depression and noticed significantly decreased P3 amplitudes in the frontal area; besides, their response time to stimulation was also significantly slower than the healthy controls, suggesting that they have decreased attention at the initial stage and impaired frontal executive function. Werner et al^73^ employed a memory coding task in 11 young patients with unipolar depression. BOLD‐fMRI revealed increased activity of the parahippocampal gyrus and decreased activity of the prefrontal cortex and parietal lobe, indicating that depression is associated with alterations in brain memory–related function. Akashi et al^74^ investigated the changes in oxyhemoglobin levels under VFT in patients with mild depression. And 52‐channel NIRS monitoring showed significantly lower oxyhemoglobin concentration in the bilateral prefrontal cortex and temporal lobe cortex under VFT compared with healthy controls, which suggests a reduced frontotemporal lobe activation and abnormal function of the frontal and temporal cortex. Akiyama et al^75^ also found decreased oxyhemoglobin concentration in the left lateral frontal lobe and temporal lobe and visibly reduced activation under VFT in patients with major depressive disorder, indicating that the left frontal lobe has low blood oxygen metabolism. And it is believed that the left frontal dysfunction is associated with depression.

### 3.2 Exercise affects brain plasticity in patients with depression

#### 3.2.1 Exercise rebuilds brain structure

Exercise is closely related to certain brain structures, and it may affect depressive emotion by rebuilding brain structure. Animal experiments have shown that exercise can improve hippocampal structure under depression. The effects of aerobic exercise or moderate‐intensity comprehensive exercise on brain structure have been well studied in patients with depression. Chen et al^76^ gave chronic unpredictable stress stimulation to rats, and a 4‐week treadmill exercise regime (20 minutes each time) resulted in significant increases in total length and total volume of capillaries in the hippocampal dentate gyrus (DG) and CA1 regions. A recent study investigated the effect of aerobic exercise on BDNF expression in the hippocampal DG and the spatial learning and memory ability in rats with chronic stress. The results showed that rats in the exercise group had significantly increased BDNF neurons and hippocampal DG region (all *P* < .05), suggesting that structural changes in the hippocampal DG and CA1 regions may be associated with depression‐like brain structure, which may contribute to targeted exercise therapy of depression.^77^

Although animal experiments have shown that exercise can promote positive changes in brain structural morphology, a few studies have contradictorily reported that exercise has no significant effect on brain plasticity in depression. Courtright^78^ investigated the changes in hippocampal volume and N‐acetylaspartic acid (NAA) concentration after aerobic exercise in patients with moderate‐to‐severe depression (18‐24 years). The subjects underwent 12‐week running (3 times/week, target heart rate: 60%–85%) and cycling, and the Hamilton Rating Scale for Depression (HAMD) scores were decreased, but hippocampal volumes and NAA concentration showed no significant changes. Similarly, Krogh et al^79^ found no significant changes in bilateral hippocampal volume after a 3‐month aerobic exercise (3 times per week, target heart rate: 80%) in patients with mild‐to‐moderate depression. Age of onset and number of depressive episodes are important factors affecting the hippocampus structure, and the changes in neurobiological parameters often lag, which may explain these results. Depressive symptoms often occur in patients with schizophrenia; studies have shown that exercise has positive effects on the brain structure of patients with schizophrenia. Pajonk et al^80^ employed a three‐month aerobic exercise approach in 16 patients with schizophrenia (3 times per week, exercise for 30 minutes), and found that hippocampal volume increased significantly (by 12%) after exercise. Moreover, the hippocampal volume was significantly correlated with the aerobic capacity of patients (*r* = .71, *P* = .003). Another study further revealed that improved cardiopulmonary function had a positive correlation with increased bilateral ventricular volume and thickening of the frontal lobe, temporal lobe, and cingulate cortex in the left hemisphere of patients with schizophrenia. Altogether, it is suggested that exercise can protect the brain structure of patients with schizophrenia and prevent depression symptoms.^81^

#### 3.2.2 Exercise activates function of related brain regions

Exercise can promote brain function in patients with depression, affect brain function, and promote generation of positive emotions. ERP and EEG studies have revealed the differences in functional activation of brain regions associated with exercise. Many ERP studies have investigated activation of brain function by aerobic exercise in depressive patients. Olson et al^82^ recruited 50 patients with major depression and compared ERP changes under the Flanker task. Patients in the experimental group were given 8 weeks of aerobic exercise (3 times per week, exercise for 45 minutes, heart rate reserve: 40%–65%). And exercise led to N2 amplitude increase (*P* < .05) and Beck Depression Inventory‐II (BDI‐II) scores decrease (12.6%), suggesting increased cognitive control ability and improved depression symptoms after exercise. Alderman et al^83^ assigned 22 patients with major depressive disorder an 8‐week aerobic meditative exercise regime (twice a week, 1‐hour exercise, 50%–70% VO_2max_), and noticed increased N2 and P3 amplitudes, reduced BDI‐II depression scores (by 40%), and reduced Ruminative Response Scale scores in the patients, suggesting that aerobic exercises can activate cerebral cortical neuron excitability and promote nerve regeneration. In addition, a study found that mindfulness training (including both sitting and walking meditation) increased contingent negative variation (CNV) amplitudes in patients with recurrent depression, indicating that CNV of ERP is activated after exercise, which can avoid negative emotions and reduce depressive rumination.^84^

Silveira et al^85^ investigated the relationship of EEG changes with exercise in elderly patients with depression using a 6‐month aerobic exercise regime (twice a week, 40%–60% VO_2max_). Results showed that the P3, P4, T5, T6, O1, and O2 regions of the patients exhibited lower θ wave frequencies than the healthy control group. After exercising, these frequencies were increased and HAMD scores were decreased (*P* = .001), suggesting that aerobic exercise promoted excitement and improved depression by activating cerebral cortical activity. Previous studies have confirmed that Taijiquan can stimulate the central nervous system and enhance brain neuronal activity. A study investigated brainwave characteristics of patients with anxiety after Taijiquan exercises. Results showed that α1, α2, and β1 rhythms were increased in both high and low anxiety groups, indicating that anxiety has been converted into mind and body relaxation and emotional stability after Taijiquan exercises.^86^ Moreover, Chan et al^87^ compared the effects of cognitive behavioral therapy and meditation on patients with depression based on a traditional Zen approach. The authors found that EEG lateralization level in the frontal lobes was increased after meditation therapy, with the left frontal hemisphere region becoming consistent with θ waves. Other groups showed no obvious changes, suggesting that the Zen exercises could activate positive emotions and improve the attention of patients with depression.

In summary, appropriate exercise can induce positive changes in EPR and EEG, and activate brain function in patients with depression. However, related studies on exercise and neuroimaging in depression are rarely reported. The related studies should be strengthened, and exercise‐induced activation of depression‐related brain regions should be comprehensively examined.

#### 3.2.3 Exercise promotes adaptive behavioral changes

Patients with depression are characterized by a decline or damage in execution, attention, and memory abilities. Proper exercise can help to improve these behavioral functions and the ability to cope with the depressive emotion. Thirty patients with depression were divided into a yoga group (8 weeks, 3 times per week) and a conventional drug treatment group; all participants completed a letter cancelation test (LCT), trail making test (TAT), forward digital span (FDS) test, and reverse digital span (RDS) test. The results showed that LCT, TTA, and TTB were improved in both groups, while LCT and RDS were significantly improved only in the yoga group (*P* < .05), indicating that yoga can benefit the attention and memory of depressive patients. In addition, the author(s) also believed that yoga can improve behavioral function after depression, mainly by regulating the limbic system and hypothalamic‐pituitary‐adrenal axis.^88^

Vasques et al^89^ studied the depressive patients who were over 70 years old; the participants were given aerobic exercise with a maximum heart rate of 65%–75% for 30 minutes. The patients had significantly improved executive function (*P* < .05) immediately after exercise, but showed no significant difference in the digit span test. At 15 minutes after exercise, only the executive function showed significant difference compared with before exercise and immediately after exercise. Viola et al^90^ found that 4‐week aerobic exercise (3 times a week and 45 minutes each time) could improve the working memory, processing speed, visual learning function, and psychopathological symptoms of depressive patients. Luttenberger et al^91^ investigated the effect of a new exercise (ie, bouldering) on behavioral function of depressive patients, and noticed that patient had significantly increased emotional processing ability compared with the control group (*P* = .010), but with no significant difference in attention levels between the two groups. The authors concluded that bouldering may serve as a new exercise treatment for depression.

Depression can lead to brain structure changes and affect brain function. Exercise can effectively protect brain plasticity and promote brain health. Furthermore, appropriate exercise has positive impact on maintaining the integrity of hippocampal volume and white matter volume, promoting the regeneration of hippocampus, activating the function of prefrontal cortex, and eventually improving the brain neuroprocessing efficiency and delaying cognitive degradation in depression patients.

## 4 SUMMARY AND PROSPECTS

### 4.1 Summary

A number of factors, including biological, psychological, and social environments, are involved in the development of depression. Techniques such as neuroimaging approaches have greatly promoted the studies on brain plasticity in depression. In recent years, the effects of exercise on depression and the resultant brain plasticity have become a research focus and there have been important findings:

1. Epidemiological studies have shown that increased exercise load is associated with reduced risk of depression.
2. Aerobic exercise, resistance exercise, and mind‐body exercise can alleviate depressive symptoms and lower depression levels, which suggests that different exercise patterns can be adopted according to different patients. The intensity and long‐term effect of exercise have become topical research issues.
3. Depression patients have different degrees of impairment in brain structure and function, as reflected in the changes in hippocampal structure, frontal lobe, temporal lobe, cerebellum, and other regional functions. A variety of neuroimaging techniques (represented by BOLD‐fMRI and NIRS) and neuroelectrophysiological techniques (represented by EEG and ERP) have revealed brain plasticity changes in depression.
4. Exercise has a positive effect on brain plasticity in patients with depression. It can rebuild brain structure, activate related brain regions, and promote adaptive changes in behavior; it also has a positive effect on maintaining hippocampal volume and white matter volume integrity, thus improving brain nerve processing efficiency and delaying degradation of cognitive function. The neuroprotective and brain activation effects of exercise have been revealed from a mechanistic perspective.

### 4.2 Future research prospects

#### 4.2.1 To establish accurate exercise prescriptions for depressive symptoms

Researchers have designed many exercise intervention programs, but the existing studies are mainly based on the recommendations of the American Sports Medicine Society, and consider only the general functions of exercises, so they are not fully applicable for depressive population. Besides, the relevant intensities of aerobic exercise, resistance exercise, and mind‐body exercise are not clearly defined. And the specific mechanisms of different exercise patterns against depression remain unclear. Therefore, the brain plasticity of different exercise patterns needs to be further clarified, and the load, frequency, and duration of exercises should be seriously designed to formulate more accurate exercise prescriptions.

#### 4.2.2 To emphasize studies of different depressive populations

Existing research has mainly focused on depressive adults around 60 years old. With the increase in age, brain plasticity and cognitive function will decline; therefore, it is easier to judge the effects of exercise interventions. However, fewer studies have focused on young people and children. At present, depression is more frequently found in younger people, with the incidence in adolescents being 5%–8%. Children and adolescents are in a sensitive period of mind‐body development, which gives it especial significance to prevent and treat depression through exercise. Future studies on antidepression exercise should consider different age groups.

#### 4.2.3 To combine multimodal brain imaging with multiple analytical methods

Single‐modality and single‐analysis methods have limitations in identifying the brain plasticity mechanisms underlying the involvement of exercise in depression. Multimodal brain imaging combined with multiple analytical methods can approach the effect of exercise on brain structure and function in a more comprehensive way and from different aspects. In future studies, techniques such as fMRI, VBM, ERP, EEG, and NIRS should be used comprehensively to collect data, eventually contributing to the early diagnosis and exercise intervention of depression.

## CONFLICT OF INTEREST

The authors declare no conflict of interest.
